# Employment, volunteering, and health‐related resource use in pre‐symptomatic AD: Results from the Anti‐Amyloid Treatment in Asymptomatic Alzheimer's Disease (A4) study

**DOI:** 10.1002/alz.70641

**Published:** 2025-10-14

**Authors:** Carolyn W. Zhu, Charlene Flournoy, Rema Raman, Mary Sano

**Affiliations:** ^1^ Brookdale Department of Geriatrics and Palliative Medicine Icahn School of Medicine at Mount Sinai New York New York USA; ^2^ Alzheimer Disease Research Center Department of Psychiatry Icahn School of Medicine at Mount Sinai New York New York USA; ^3^ James J Peters VA Medical Center Bronx New York USA; ^4^ Alzheimer's Therapeutic Research Institute University of Southern California San Diego California USA

**Keywords:** amyloid, dementia, employment, health‐related resources, pre‐symptomatic, productive time use

## Abstract

**INTRODUCTION:**

Little is known about productive time use and health‐related resource use during “pre‐symptomatic” AD, defined by the presence of brain amyloid in the absence of cognitive symptoms. We compared changes in resource use and participation in paid employment and/or volunteering in cognitively unimpaired older adults with amyloid accumulation (Anti‐Amyloid Treatment in Asymptomatic Alzheimer's Disease [A4] study, *N* = 1165) to otherwise matched participants without amyloid accumulation (Longitudinal Evaluation of Amyloid Risk and Neurodegeneration [LEARN] study, *N* = 507).

**METHODS:**

Health‐related resource use was self‐reported using the Resource Use Inventory (RUI). Longitudinal analyses examined effect on RUI from study (A4 vs LEARN), time, and their interaction, controlling for Alzheimer's Disease Cooperative Study‐Preclinical Alzheimer's Cognitive Composite (ADCS‐PACC) and the Clinical Dementia Rating (CDR) scale, and their change scores from baseline.

**RESULTS:**

Over time, paid employment and volunteering decreased, and unpaid help and hospitalization increased. Results showed clear associations between ADCS‐PACC and CDR with RUI.

**DISCUSSION:**

Little detectable impact of amyloid levels on RUI was found in pre‐symptomatic AD that has been identified as an ideal stage to target for dementia prevention.

**Highlights:**

- Using data from a cohort of cognitively unimpaired older adults with evidence of amyloid accumulation enrolled in the Anti‐Amyloid Treatment in Asymptomatic Alzheimer's Disease (A4) study and otherwise matched participants who did not meet subthreshold levels of amyloid accumulation enrolled in the Longitudinal Evaluation of Amyloid Risk and Neurodegeneration (LEARN) study, this study showed clear associations between clinical variables and resource use and participation in paid employment and volunteering but suggested little detectable impact of amyloid levels on rate of change during the preclinical stage.
- Our results suggest that economic benefits from currently available treatment that effectively removes amyloid may not be immediately or concurrently observed during the short timeline of clinical trials.
- It is critical that our examination of economic consequence of treatment include broad ranges of items on resource use and productivity loss, longer time horizon, and that we balance between cost of detection, treatment, and burden and benefit.

## 1 INTRODUCTION

Although the costs of dementia are vast and continue to grow,^1^ even mild cognitive loss is associated with increased costs of care. There is evidence of increased health care utilization and costs around the onset of clinical symptoms when patients begin to experience early‐stage mild cognitive impairment (MCI) or symptomatic dementia^2^ and a year before receiving a diagnosis of MCI^3^. Approximately 1 in 5 patients with MCI (20.6%) and 1 in 10 patients with mild Alzheimer's disease (AD; 11.1%) work for pay,^4^ suggesting that costs associated with patient productivity losses are also important. With few exceptions, studies to date have not examined patient productivity. One study estimated AD patient's own market productivity loss at $7937 and non‐market productivity (e.g., volunteering) at $10,785 per person per year.^5^ In patients with MCI or mild dementia who are often targeted for AD trials, faster rate of decline from baseline has been shown to be independently associated with a lower likelihood of employment/volunteer work.^6^

With the advent of biomarkers that can detect disease pathology prior to symptom onset, there is urgent need to understand the value of pre‐symptomatic diagnoses^7^, ^8^ and disease‐modifying treatments (DMTs). ^9^, ^10^ “Pre‐symptomatic” AD, defined by the presence of brain amyloid in the absence of cognitive symptoms, has been identified as an ideal stage to target for the prevention of progression to cognitive impairment and dementia. However, few studies have examined resource use and costs associated with pre‐symptomatic AD.^4^, ^11^, ^12^ Using baseline data from the Longitudinal Cohort Study of Resource Use and Cost of Mild Cognitive Impairment and Mild Dementia Due to Alzheimer's Disease in the United States (GERAS‐US) in which 52% of the study participants were confirmed as amyloid positive, Robinson et al. reported higher costs associated with participant's cognitive and functional status but not with amyloid status.^4^, ^11^ Chandler and Kubisiak identified a small cohort of GERAS‐US participants who had Medicare Fee‐for‐Service and showed a statistically significant lower cost in amyloid‐positive than in amyloid‐negative mild AD, which the study attributed to higher comorbidity profile in amyloid‐negative AD in their study.^4^ Separately, data from the Swedish registry for cognitive/dementia disorders (SveDem) showed lower annual costs in amyloid‐positive compared to amyloid‐negative individuals.^12^ None of these studies included the patient's own productivity loss as part of cost estimates.

The Anti‐Amyloid Treatment in Asymptomatic Alzheimer's Disease (A4) study (ClinicalTrials.gov ID: NCT02008357) was a 240‐week, Phase 3 double‐blind, placebo‐controlled randomized clinical trial of solanezumab, a humanized monoclonal antibody targeting the mid‐peptide domain of amyloid beta (Aβ), in cognitively unimpaired adults 65–85 years of age with evidence of amyloid accumulation.^13^ Although the study did not show slowed progression of preclinical AD between drug and placebo groups, the A4 study provides a rich data source for understanding cognitive and functional decline at the preclinical stage. In particular, the Resource Use Inventory (RUI),^14^ designed as a self‐administered questionnaire to be completed by study participant and/or partner to assess health‐related service utilization and time use, was administered as a secondary outcome. A natural history observational arm of the A4 study, the Longitudinal Evaluation of Amyloid Risk and Neurodegeneration (LEARN) study (ClinicalTrials.gov ID: NCT02488720), comprised individuals who matched A4 participants on all screening criteria, but screen failed due to subthreshold levels of amyloid accumulation, was run in parallel with identical cognitive assessments. The LEARN cohort serves as an essential comparison group to quantify amyloid‐related cognitive decline and also provides data on non‐amyloid factors associated with cognitive decline.

In this study, we sought to fill a gap in the literature on resource use in pre‐symptomatic AD, including patients’ productivity loss, and examining resource use by patients’ amyloid status. We describe changes in health‐related resource use over time in the A4/LEARN “pre‐symptomatic” cohorts and examine the effects of amyloid burden and clinical characteristics. We focus on two clinical characteristics that are of particular importance: (1) the ADCS‐PACC (Alzheimer's Disease Cooperative Study‐Preclinical Alzheimer's Cognitive Composite)^15^ because it is the primary outcome for the A4 study, and (2) the Clinical Dementia Rating (CDR) scale^16^ because it has been most commonly used as an endpoint in AD clinical trials.^17^

## 2 METHODS

### 2.1 A4 and LEARN study design

The A4 study protocol and methods have been published.^13^, ^18^ Here we describe relevant aspects of the current analysis. Briefly, the A4 study was a 240‐week, Phase 3 double‐blind, placebo‐controlled randomized clinical trial of solanezumab, a humanized monoclonal antibody targeting the mid‐peptide domain of Aβ, in non‐demented adults 65–85 years of age with evidence of amyloid accumulation. Specifically, individuals with CDR global scale score of 0, Mini‐Mental State Examination (MMSE) score of 25–30, and Logical Memory Delayed Recall (LMDR IIA) score of 6–18 were eligible. The double‐blind phase of the trial was conducted over 240 weeks with an open‐label extension to 312 weeks. The study did not show slowed progression of preclinical AD between treatment and placebo groups. At the same time, a natural history observational arm of the A4 study, the LEARN study, comprised individuals who matched A4 participants on all screening criteria, but screen failed due to subthreshold levels of amyloid accumulation, was run in parallel with identical assessments. All participants were required to have a study partner who was familiar with their functioning and consented to participate in data collection.

All participants received a baseline florbetapir positron emission tomography (PET) scan, an annual CDR evaluation, and cognitive testing every 6 months with the ADCS‐PACC and MMSE during the study.

### 2.2 Health‐related resource use

Health‐related resource use was self‐reported by participant and/or study partner using the RUI^14^ administered as a secondary outcome. The RUI consists of questions that aim to reflect the most important aspect of direct medical care (hospitalizations, doctor's visits), paid care, and unpaid care provided by family and friends. In addition to these traditional measures of health‐related resource use items, the RUI also collects data on participants’ time spent in paid employment and volunteer work. The RUI has been used in AD prevention trials and shown to differentiate between MCI and cognitively normal individuals, and it is useful for tracking resource and time use through transition from healthy to cognitive impairment.^14^, ^19^ Recall interval for hospitalizations was 1 year. For other items, participants and their study partners were asked to recall utilization during the past 3 months.

**RESEARCH IN CONTEXT**

1. **Systematic review**: “Pre‐symptomatic” Alzheimer's disease (AD), defined by the presence of brain amyloid in the absence of cognitive symptoms, has been identified as an ideal stage to target for the prevention of progression to cognitive impairment and dementia. However, little is known about health economic outcomes during this disease stage, including an individual's ability to work and/or volunteer and health‐related resource use.
2. **Interpretation**: Our findings showed clear associations between clinical variables and resource use and participation in paid employment and volunteering but suggested little detectable impact of amyloid levels on rate of change during the preclinical stage.
3. **Future directions**: Results suggest that economic benefits from currently available treatment that effectively removes amyloid may not be immediately or concurrently observed during the short timeline of clinical trials. Our examination of economic consequence of treatment should include broad ranges of items, longer time horizon, and balance between cost of detection, treatment, burden, and benefit.

### 2.3 Amyloid PET

Brain amyloid was assessed with 18F‐Florbetapir PET using a mean cortical imaging standardized uptake value ratio (SUVR).^20^ Elevated amyloid was primarily defined by a SUVR threshold of 1.15, a SUVR of 1.10 to <1.15 was considered elevated only when a visual read was deemed positive by a two‐reader consensus determination. SUVRs were converted to Centiloids (CL) on a scale of 0 to 100.

### 2.4 Clinical assessments

We focus on two clinical characteristics that are of particular importance. The primary outcome for the A4 study, the ADCS‐PACC, ^15^ is a composite of well‐validated neuropsychological tests selected specifically for their sensitivity in tracking the earliest evidence of decline from normal cognition to subtly abnormal cognitive performance. It includes the MMSE, Digit Symbol Substitution Test from the Wechsler Adult Intelligence Scale–Revised, Delayed Recall score on the Logical Memory IIa sub‐test from the Wechsler Memory Scale, and Total Recall score from the Free and Cued Selective Reminding Test. The ADCS‐PACC composite score is determined from its components using an established normalization method^21^ and has been validated previously.^15^ A higher score indicates better cognitive performance.

The CDR scale^16^ is an integrated measure of both cognition and function in AD that has been commonly used as an endpoint in AD clinical trials.^17^ The CDR assesses on an ordinal scale (ranging from 0 to 3, from no impairment to severe impairment) in six domains consisting of memory, orientation, judgment/problem solving, community affairs, home and hobbies, and personal care, termed “box scores.” The CDR Sum of Boxes (CDR‐SB) score is constructed by adding the domain scores to yield a continuous variable, ranging from 0 (no impairment in any of the boxes) to a maximum value of 18 (severe impairment with a score of 3 in all six domain boxes).

Although comorbidities data have been collected in the study, they were not approved for data sharing at this time.^22^ We used available data on vital signs and constructed an indicator for hypertension, defined by World Health Organization definition of systolic blood pressure ≥140 mmHg and/or diastolic pressure ≥90 mmHg. Body mass index (BMI) was computed using height and weight measured at baseline.

### 2.5 Demographic characteristics

Demographic characteristics collected at the initial screening visit included self‐reported age, sex, race (“American Indian or Alaska Native,” “Asian,” “Black or African American,” “More than one race” “Unknown or not reported,” and “White”), ethnicity (“Hispanic or Latino,” “Not Hispanic or Latino,” and “Unknown or Not Reported”) using National Institutes of Health (NIH) definitions and years of education. Because of small numbers of participants from historically race and ethnic underrepresented groups (RE‐URG), an indicator variable for anybody who was not self‐identified as “White” and “Non‐Hispanic” was constructed for analyses purposes. Apolipoprotein E (*APOE*) ε4 carrier status (carrier/non‐carrier) was determined through an initial blood draw.

### 2.6 Statistical analyses

Participant characteristics were summarized using means and SDs for continuous variables and counts and percentages for categorical variables. Because the A4 study did not demonstrate slowing in cognitive or clinical outcomes between the active (solanezumab) and placebo groups, the analyses combined the active and placebo groups of the A4 study (*N* = 1165) and compared the full A4 sample to the LEARN natural history companion study (*N* = 507). Differences between A4 and LEARN groups were compared using the *t*‐test for continuous variables and the chi‐square test for categorical variables.

To assess the effects of amyloid burden and clinical characteristics on RUI outcomes, our estimation models included independent variables of (1) study arm (A4 vs LEARN), (2) clinical characteristics (ADCS‐PACC or CDR‐SB) assessed at baseline, and (3) change from baseline in ADCS‐PACC or CDR‐SB at each visit. Time was measured as a continuous variable defined as months since baseline examination date. Multivariable mixed‐effects logistic regression models were used to examine the relationship between the independent variables and RUI items over time, controlling for age, sex, indicator for RE‐URG, education, BMI, and hypertension as fixed effects, and a random intercept and random slope for each participant. All analyses were performed using Stata 17.0.^23^ Statistical significance was set a priori at *p* < 0.05.

Main analyses were conducted for the duration of the double‐blind period of the A4 study, namely 240 weeks. We performed several supplemental analyses. First, to estimate whether the effects from baseline amyloid burden or clinical measures on RUI outcomes changed over time, we tested their interaction terms with time in exploratory models. Second, we tested inclusion of *APOE* ε4 carrier status in the models. Third, we re‐ran the models using data including open‐label phase.

Finally, because participants with SUVR between 1.10 and <1.15 could be considered having elevated amyloid and enrolled in A4 or not elevated and enrolled in LEARN study based on a visual read, instead of using study indicator to proxy amyloid burden, we explored the relationship between amyloid CL tertiles with RUI.

## 3 RESULTS

### 3.1 Participant characteristics

A4 participants were older (mean ± SD age = 71.9 ± 4.8 vs 70.5 ± 4.3 years), had lower MMSE (28.8 ± 1.3 vs 29.0 ± 1.2) at baseline, and more likely to have at least one *APOE* ε4 allele (59.0% vs 22.5%) than LEARN participants. There were no differences between A4 and LEARN in years of education (16.6 ± 2.8), sex (≈60% female), race/ethnicity (≈10% RE‐URG), BMI (27.4 ± 5.0), or hypertension (39.2%). There was no statistically significant different in RUI items A4 and LEARN except for lower rate of volunteering reported in A4 (57.9%) compared to LEARN (65.5%) (Table 1).

**TABLE 1:** Characteristics at first RUI visit.

	All		A4		LEARN		*p*
*N*	1672		1165		507		
Variable	Mean	SD	Mean	SD	Mean	SD	
Age	71.5	4.7	71.9	4.8	70.5	4.3	<0.001
Female, %	59.9		59.5		61.1		0.55
Years of education	16.6	2.7	16.6	2.8	16.8	2.6	0.195
RE‐URG, %	9.5		9.1		10.3		0.467
Any *APOE* ε4, %	47.9		59.0		22.5		<0.001
MMSE	28.9	1.3	28.8	1.3	29.0	1.2	<0.001
ADCS‐PACC	0.27	2.6	0.01	2.67	0.87	2.35	<0.001
**CDR‐SB**	0.05	0.2	0.05	0.15	0.05	0.15	0.361
Systolic blood pressure	136.7	(16.0)	136.6	(16.1)	136.9	(16.0)	0.584
Diastolic blood pressure	76.2	(9.4)	76.1	(9.5)	76.6	(9.2)	0.154
BMI	27.4	(5.0)	27.4	(5.1)	27.6	(4.9)	0.148
Hypertension (%)	39.2		38.3		41.4		0.229
**Any utilization of RUI items, %**							
Hospitalization	7.6		7.3		8.5		0.424
Doctor visit	78.4		77.6		80.3		0.244
Paid help	1.3		1.2		1.6		0.641
Unpaid help	2.5		2.1		3.2		0.230
Any paid or unpaid help	3.4		2.8		4.5		0.078
Paid employment	29.1		29.5		28.2		0.584
Volunteering	60.2		57.9		65.5		0.003
Any employment or volunteer work	72.8		71.6		75.5		0.095

### 3.2 Multivariable analyses results on the relationships between A4/LEARN group, ADCS‐PACC, and RUI

Multivariable analyses results showed that when controlling for other covariates, for the entire sample, the likelihood of participation decreased each month in paid employment (odds ratio [OR] ± standard error [SE] = 0.960 ± 0.006) and volunteering (OR ± SE = 0.989 ± 0.003), and the likelihood of receiving unpaid help (OR ± SE = 1.026 ± 0.006) and having had a hospitalization (OR ± SE = 1.009 ± 0.004) increased (Table 2). At baseline, A4 participants were more likely than LEARN participants to engage in paid employment (OR ± SE = 2.050 ± 0.608) but less likely to volunteer (OR ± SE = 0.460 ± 0.106), and less likely to have had a hospitalization (OR ± SE = 0.653 ± 0.092). Results from Table S1 show that none of the interaction terms between study arm (A4 vs LEARN) and time were statistically significant, suggesting that there was no difference in the rate of change in participation in employment/volunteering or use of health‐related resources over time between the two groups.

**TABLE 2:** Relationship between Study Arm, ADCS‐PACC, and RUI.

Variables	Employment	Volunteer	Paid help	Unpaid help	Hospitalization	Doctor visit
	OR (SE)	OR (SE)	OR (SE)	OR (SE)	OR (SE)	OR (SE)
Time, months since baseline	0.960^***^	0.989^**^	1.005	1.026^***^	1.009^*^	0.998
	(0.006)	(0.003)	(0.010)	(0.006)	(0.004)	(0.003)
Baseline group difference, A4 vs LEARN	2.050^*^	0.460^***^	0.761	0.888	0.653^**^	0.940
	(0.608)	(0.106)	(0.258)	(0.198)	(0.092)	(0.101)
Baseline ADCS‐PACC	1.107^†^	1.168^***^	0.934	0.995	0.960^†^	1.030
	(0.064)	(0.052)	(0.056)	(0.038)	(0.024)	(0.023)
ADCS‐PACC change from baseline	1.058	1.183^***^	0.862^*^	0.926^†^	0.981	1.086^***^
	(0.054)	(0.041)	(0.058)	(0.038)	(0.027)	(0.023)
Age	0.776^***^	1.100^***^	1.101^**^	1.112^***^	1.045^***^	1.019
	(0.026)	(0.026)	(0.033)	(0.027)	(0.014)	(0.012)
Female vs male	0.131^***^	1.684^*^	1.710^†^	2.417^***^	0.903	0.915
	(0.044)	(0.372)	(0.518)	(0.538)	(0.117)	(0.093)
RE‐URG vs NHW	5.527^**^	0.583	0.597	1.069	0.819	0.868
	(3.236)	(0.200)	(0.334)	(0.323)	(0.185)	(0.141)
Education	1.167^**^	1.203^***^	1.125^*^	1.047	1.011	1.041^*^
	(0.068)	(0.048)	(0.053)	(0.036)	(0.027)	(0.020)
BMI	1.024	0.979	1.114^***^	1.042^*^	1.052^***^	1.030^**^
	(0.026)	(0.020)	(0.034)	(0.021)	(0.012)	(0.010)
Hypertension	1.382	1.494	0.499	0.719	0.693	1.371
	(0.822)	(0.638)	(0.357)	(0.327)	(0.205)	(0.289)

Interaction between group and time was not statistically significant and was dropped from the final model.

Abbreviations: BMI, body mass index; NHW, non‐hispanic white; RE‐URG, Race/ethnic under representative group

* *p* < 0.05.

** *p* < 0.01.

*** *p* < 0.001.

^†^ *p* < 0.10.

At baseline, a one‐point increase in baseline ADCS‐PACC (better cognition) was associated with a higher likelihood of participating in employment (OR = 1.107 ± 0.064), higher likelihood of volunteering (OR = 1.168 ± 0.052), and lower likelihood of having a hospitalization (OR = 0.960 ± 0.024). A one‐point increase in ADCS‐PACC change score from baseline (i.e., improvement in cognition) was associated with a higher likelihood of volunteering (OR = 1.183 ± 0.041), lower likelihood of receiving paid (OR = 0.862 ± 0.058) and unpaid (OR = 0.926 ± 0.038) care, and higher likelihood of having a doctor's visit (OR = 1.086 ± 0.023).

Relationships between RUI items and participant demographic characteristic were as expected. Specifically, older age was associated with a lower likelihood of participating in paid employment (OR ± SE = 0.776 ± 0.026) but a higher likelihood of volunteering (OR ± SE = 1.110 ± 0.026), higher likelihood of receiving paid (OR ± SE = 1.101 ± 0.033) and unpaid help (OR ± SE = 1.112 ± 0.027), and higher likelihood of having a hospitalization (OR ± SE = 1.045 ± 0.014). Women were less likely than men to participate in paid employment (OR ± SE = 0.131 ± 0.044), but more likely to volunteer (OR ± SE = 1.684 ± 0.372), and more likely to receive unpaid help (OR ± SE = 2.417 ± 0.538). RE‐URG was associated with a higher likelihood of participating in paid employment (OR ± SE = 5.521 ± 3.236). Higher education was associated with a higher likelihood of participating in paid employment (OR ± SE = 1.167 ± 0.068), volunteering (OR ± SE = 1.203 ± 0.048), and receiving paid help (OR ± SE = 1.125 ± 0.053). Higher BMI was associated with a higher likelihood of receiving paid (OR ± SE = 1.114 ± 0.034) and unpaid help (OR ± SE = 1.042 ± 0.021), having a hospitalization (OR ± SE = 1.052 ± 0.012), and having a doctor's visit (OR ± SE = 1.030 ± 0.010).

### 3.3 Multivariable analyses results on the relationships between A4/LEARN group, CDR‐SB, and RUI

We also estimated relationships with RUI items using CDR‐SB instead of ADCS‐PACC scores (Table 3). Results showed that when controlling for other variables, compared to those with CDR‐SB = 0, participants with CDR‐SB = 0.5 were less likely to participate in paid employment (OR = 0.393 ± 0.164) or volunteer (OR = 0.378 ± 0.143) at baseline. A one‐point increase in the change in CDR‐SB from baseline (i.e., worsening) was associated with lower likelihood of employment (OR = 0.636 ± 0.137), volunteering (OR = 0.558 ± 0.082), and a higher likelihood of paid (OR = 1.553 ± 0.204) and unpaid (OR = 1.312 ± 0.104) care. At baseline, A4 participants were more likely than LEARN participants to engage in paid employment (OR ± SE = 2.442 ± 0.745) but less likely to volunteer (OR ± SE = 0.395 ± 0.102), and less likely to have had a hospitalization (OR ± SE = 0.746 ± 0.112). Coefficient estimates from other independent variables were consistent with those using ADCS‐PACC scores. Results from Table S2 show that the interaction terms between study arm and time, and between baseline CDR‐SB and time was not statistically significant, suggesting that study indicator as a measure of amyloid burden and baseline CDR‐SB waws not associated with the rate of change over time in the RUI outcomes.

**TABLE 3:** Relationship between study arm, CDR‐SB, and RUI.

Variables	Employment	Volunteer	Paid help	Unpaid help	Hospitalization	Doctor visit
	OR (SE)	OR (SE)	OR (SE)	OR (SE)	OR (SE)	OR (SE)
Time, months since baseline	0.953^***^	0.995	1.002	1.023^**^	1.009^†^	0.993^†^
	(0.007)	(0.005)	(0.012)	(0.008)	(0.005)	(0.004)
Baseline group difference, A4 vs LEARN	2.442^**^	0.395^***^	0.925	0.895	0.746^†^	0.953
	(0.745)	(0.102)	(0.326)	(0.200)	(0.112)	(0.110)
Screening CDR‐SB = 0.5 vs 0	0.393^*^	0.378^**^	0.999	1.106	1.120	1.402^†^
	(0.164)	(0.143)	(0.443)	(0.326)	(0.216)	(0.243)
CDR‐SB change from baseline	0.636^*^	0.558^***^	1.553^***^	1.312^***^	1.072	1.047
	(0.137)	(0.082)	(0.204)	(0.104)	(0.086)	(0.088)
Age	0.777^***^	1.064^*^	1.157^***^	1.126^***^	1.053^***^	1.001
	(0.026)	(0.027)	(0.034)	(0.025)	(0.014)	(0.011)
Female vs male	0.152^***^	2.113^**^	1.722^†^	2.078^***^	0.928	0.898
	(0.050)	(0.512)	(0.538)	(0.422)	(0.119)	(0.094)
RE‐URG vs NHW	5.085^**^	0.585	0.669	1.009	0.988	0.970
	(2.813)	(0.228)	(0.377)	(0.309)	(0.210)	(0.167)
Education	1.228^***^	1.238^***^	1.158^**^	1.063^†^	0.979	1.035^†^
	(0.074)	(0.056)	(0.055)	(0.037)	(0.025)	(0.020)
BMI	1.010	0.969	1.114^***^	1.058^**^	1.060^***^	1.029^**^
	(0.025)	(0.022)	(0.030)	(0.018)	(0.012)	(0.011)
Hypertension	0.902	1.124	0.170^†^	0.711	1.009	1.279
	(0.530)	(0.552)	(0.161)	(0.281)	(0.267)	(0.277)

Interaction between group and time was not statistically significant and was dropped from the final model.

* *p* < 0.05.

** *p* < 0.01.

*** *p* < 0.001.

^†^ *p* < 0.10.

### 3.4 Supplemental analyses on relationships between amyloid Centiloid (CL) and RUI

Depending on a visual read from a two‐reader consensus, participants with SUVR between 1.10 and <1.15 could be considered as having elevated amyloid and enrolled in the A4 study or not elevated and enrolled in LEARN study. Instead of using study indicator to proxy amyloid burden, we explored the relationship between amyloid CL tertiles with RUI. Results show that regardless of clinical variables, amyloid CL tertiles were not associated with RUI items at baseline or rate of change in RUI over time (Tables S3,S4).

### 3.5 Additional supplemental analyses

To estimate whether the effects from baseline amyloid burden or clinical measures on RUI outcomes changed over time, we included their interaction terms with time in exploratory models. None of the interaction terms were statistically significant and did not affect the main results. Models including *APOE* ε4 carrier status were not substantively different from the main results. Data that included open‐label phase were similar to the main results. Results from these analyses are available upon requests.

## 4 DISCUSSION

Amyloid and tau PET imaging and blood biomarkers are being used increasingly to identify older individuals in preclinical stages of AD. However, resource use and productivity loss in individuals with preclinical AD is yet to be quantified. In this study, we compared changes in health‐related resource use and economic outcomes in cognitively unimpaired older adults with evidence of amyloid accumulation, that is, pre‐symptomatic AD, to otherwise matched participants who did not meet subthreshold levels of amyloid accumulation. We included use of direct medical care, direct non‐medical care, and paid and unpaid care that have been included traditionally in cost studies. Because many cognitively unimpaired older adults continue to work for pay and/or volunteer their time productively,^24^ we also examined participation in employment and volunteering. We note several main results from our analyses: (1) Subtle decreases in the rate of employment and volunteering and increases in the likelihood of using unpaid care and hospitalization over 3–5 years were captured by the RUI. (2) Results showed clear associations between clinical observations (ADCS‐PACC, CDR‐SB) and health‐related resource use. (3) There were little detectable relationships between amyloid levels and the rate of change over time in paid employment, volunteering, and health‐related resource use at preclinical stage.

A recent study on the A4 and LEARN study data showed that higher baseline levels of amyloid and tau markers were associated with faster rates of cognitive and functional impairment.^25^ At the beginning of the study, average ADCS‐PACC scores in LEARN were 0.9 points higher than in A4. By the end of the double‐blinded phase of the study at 240 weeks (≈4.5 years), this difference between LEARN and A4 participants had grown to about 2 points in A4 participants who were in the middle tertile amyloid PET and about 5 points in A4 participants who were in the highest tertile amyloid PET. Across the LEARN and A4 cohorts, amyloid PET and phosphorylated tau‐217 (p‐tau217) markers explained much of the variance in cognitive decline. In particular, p‐tau217 consistently made a large unique contribution to explaining the variations in the rate of decline in ADCS‐PACC. However, unique contribution from amyloid PET to explaining the variations in the rate of decline in ADCS‐PACC was negligible. Independent of these biomarkers, baseline ADCS‐PACC scores also made unique contribution to explaining the variations in the rate of decline in ADCS‐PACC. These findings suggest that AD biomarkers are useful for predicting cognitive decline in an older asymptomatic population.

We did not find any studies that have examined the relationships between AD biomarkers such as amyloid status and health‐related resource use in pre‐symptomatic AD. However, our results are consistent with those from several studies on early symptomatic AD, which showed mostly statistically insignificant relationship between amyloid status and cost of care.^4^, ^11^, ^12^ One study showed a statistically significant lower cost in amyloid‐positive than in amyloid‐negative patients with mild AD, which the study attributed to worse comorbidity profile in those who were amyloid negative in their study.^4^ Because comorbidities data are not yet available at this time, we were unable to examine the comorbidity profiles and their relationships with resource use in our current study. Given the increasing focus on pre‐symptomatic AD, clearly more studies are needed to examine this relationship between amyloid and other biomarker status with health‐related resource use.

Our work adds to the small number of studies that examined patients’ own productive time use. Compared to ≈20% of patients with MCI who reported working for pay,^26^ substantially higher proportion of patients in our cohort of asymptomatic individuals reported working for pay (30%) and even more reported volunteering their time (58%). It is worth noting that the average age of MCI cohort in the earlier study and the LEARN cohort in our study was similar at ≈70 years. Although the A4 cohort in our study was slightly older than the LEARN cohort, with an average age of 71 years, there was no difference between the A4 and LEARN cohort in participation in paid work, although participation in volunteering was lower. In an earlier study in patients with MCI or mild dementia, who are often targeted for AD trials, the likelihood of employment/volunteer work was strongly associated with rate of decline in cognition and function from baseline, suggesting potential reductions in patients’ productivity loss from effective treatment that reduces the rate of decline in clinical outcomes.^6^ Similarly, we found in the current study that worse cognition at baseline and change scores from baseline were associated with lower participation rate in both paid employment and volunteering, suggesting an overall loss of productive time associated with cognitive loss. It is worth noting that there are differences in the effects of age and demographic subgroups in participation in paid work and volunteering, suggesting that time spent on paid work and volunteering should be examined separately.

Our study has several limitations. First, participants in the A4 and LEARN studies were research volunteers for clinical trials aiming to prevent cognitive decline in preclinical AD.^25^ They were in general good health, willing to commit to multiple visits over several years, and had higher rates of family history of dementia; they may not represent a generalizable population. Both A4 and LEARN studies experienced prolonged disruption of activities because of social distancing measures due to the coronavirus disease 2019 (COVID‐19) pandemic, which may have affected participants’ resource use and participation in employment and volunteering. However, there is no reason to believe such disruptions may have affected A4 and LEARN participants differently and may have led to biased estimates on their relationship with the outcomes.

## 5 CONCLUSION

Taken together, results from this study suggest that economic benefits from currently available treatment that effectively removes amyloid may not be immediately or concurrently observed during the short timeline of the clinical trial period. The benefits of using amyloid as a surrogate for disease may have limited use in assessing economic outcomes in the short term. However, in the long term, beyond the duration of clinical trials, benefits in health‐related resource use and productivity loss from current treatment that are expected to reduce the rate of decline in clinical outcomes may accrue as the effects on clinical outcomes become more pronounced. With the recent advances in prevention strategies, diagnostic methods, and DMTs in earlier stages of disease, policymakers, payers, and patients and their families, and all who are affected will require evidence on how clinical outcomes assessed in trials translate to meaningful outcomes, including quality of life, independence, resource use, and costs. It is critical that in our examination of economic consequence of treatment include broad ranges of items on resource use and productivity loss, longer time horizon, and balance between cost of treatment, detection, and burden and benefit.

## CONFLICT OF INTEREST STATEMENT

Carolyn W. Zhu reports receiving research support from the National Institute on Aging. Charlene Flournoy reports receiving research support from the National Institute on Aging and Eisai. Rema Raman reports receiving research support from the National Institute on Aging, Alzheimer's Association, American Heart Association, Eisai, and Eli Lilly; travel support from the Alzheimer's Association; and payment for Data and Safety Monitoring Board (DSMB) activity from the National Institutes of Health. Mary Sano reports receiving research support from the National Institute on Aging, NovoNordisk, Novartis, Eisai, and Axsome; and payment for DSMB activity from studies sponsored by the University of Colorado and Ohio State University. Author disclosures are available in the Supporting Information.

## Supporting information



Supporting file1:alz70641‐sup‐0001‐disclosure.pdf

Supporting file2:alz70641‐sup‐0002‐tables.docx

## References

[1] United States Cost of Dementia Research Team . The Cost of Dementia in 2025. 2025. https://schaeffer.usc.edu/research/the‐cost‐of‐dementia‐in‐2025/

[2] El‐Hayek YH , Wiley RE , Khoury CP , et al. Tip of the iceberg: assessing the global socioeconomic Costs of Alzheimer's disease and related dementias and strategic implications for stakeholders. J Alzheimers Dis. 2019;70(2):323‐341. doi:10.3233/JAD-190426 31256142 PMC6700654

[3] Lin PJ , Zhong Y , Fillit HM , Chen E , Neumann PJ . Medicare expenditures of individuals with Alzheimer's disease and related dementias or mild cognitive impairment before and after diagnosis. J Am Geriatr Soc. 2016;64(8):1549‐1557. doi:10.1111/jgs.14227 27295430

[4] Chandler J , Kubisiak J . Clinical and economic assessment in early‐stage dementia by severity and amyloid‐beta status: a 5‐year retrospective claims study of GERAS‐US patients. J Alzheimers Dis. 2023;91(2):753‐765. doi:10.3233/JAD-220415 36502319 PMC9912735

[5] Fox J , Mearns ES , Li J , et al. Indirect costs of Alzheimer's disease: unpaid caregiver burden and patient productivity loss. Value Health. 2025;28(4):519‐526. doi:10.1016/j.jval.2024.10.3851 39571734

[6] Zhu CW , Sano M . Meaningful benefit of disease‐modifying treatment: evaluating changes in health‐related resource use. Alzheimers Dement (N Y). 2024;10(3):e12455. doi:10.1002/trc2.12455 39086734 PMC11289728

[7] Janelidze S , Bali D , Ashton NJ , et al. Head‐to‐head comparison of 10 plasma phospho‐tau assays in prodromal Alzheimer's disease. Brain. 2023;146(4):1592‐1601. doi:10.1093/brain/awac333 36087307 PMC10115176

[8] Barthelemy NR , Salvado G , Schindler SE , et al. Highly accurate blood test for Alzheimer's disease is similar or superior to clinical cerebrospinal fluid tests. Nat Med. 2024;30(4):1085‐1095. doi:10.1038/s41591-024-02869-z 38382645 PMC11031399

[9] van Dyck CH , Swanson CJ , Aisen P , et al. Lecanemab in early Alzheimer's disease. N Engl J Med. 2023;388(1):9‐21. doi:10.1056/NEJMoa2212948 36449413

[10] Sims JR , Zimmer JA , Evans CD , et al. Donanemab in early symptomatic Alzheimer disease: the TRAILBLAZER‐ALZ 2 randomized clinical trial. JAMA. 2023;330(6):512‐527. doi:10.1001/jama.2023.13239 37459141 PMC10352931

[11] Robinson RL , Rentz DM , Andrews JS , et al. Costs of early stage Alzheimer's disease in the United States: cross‐sectional analysis of a prospective cohort study (GERAS‐US)1. J Alzheimers Dis. 2020;75(2):437‐450. doi:10.3233/JAD-191212 32250304 PMC7306889

[12] Aye S , Frisell O , Zetterberg H , et al. Costs of care in relation to Alzheimer's disease severity in Sweden: a national registry‐based cohort study. Pharmacoeconomics. 2025;43(2):153‐169. doi:10.1007/s40273-024-01443-2 39485581 PMC11782292

[13] Sperling RA , Rentz DM , Johnson KA , et al. The A4 study: stopping AD before symptoms begin?. Sci Transl Med. 2014;6(228):228fs13. doi:10.1126/scitranslmed.3007941 PMC4049292 24648338

[14] Sano M , Zhu CW , Whitehouse PJ , et al. ADCS Prevention Instrument Project: pharmacoeconomics: assessing health‐related resource use among healthy elderly. Alzheimer Dis Assoc Disord. 2006;20(4):S191‐202. Suppl 3. 17135812 10.1097/01.wad.0000213875.63171.87 PMC3229195

[15] Donohue MC , Sperling RA , Salmon DP , et al. The preclinical Alzheimer cognitive composite: measuring amyloid‐related decline. JAMA Neurol. 2014;71(8):961‐970. doi:10.1001/jamaneurol.2014.803 24886908 PMC4439182

[16] Hughes CP , Berg L , Danziger WL , Coben LA , Martin RL . A new clinical scale for the staging of dementia. Br J Psychiatry. 1982;140:566‐572. 7104545 10.1192/bjp.140.6.566

[17] Morris JC . The Clinical Dementia Rating (CDR): current version and scoring rules. Neurology. 1993;43(11):2412‐2414. 10.1212/wnl.43.11.2412-a 8232972

[18] Sperling RA , Donohue MC , Raman R , et al. Trial of solanezumab in preclinical Alzheimer's disease. N Engl J Med. 2023;389(12):1096‐1107. doi:10.1056/NEJMoa2305032 37458272 PMC10559996

[19] Zhu CW , Sano M , Ferris SH , Whitehouse PJ , Patterson MB , Aisen PS . Health‐related resource use and costs in elderly adults with and without mild cognitive impairment. J Am Geriatr Soc. 2013;61(3):396‐402. doi:10.1111/jgs.12132 23414481 PMC3928966

[20] Royse SK , Minhas DS , Lopresti BJ , et al. Validation of amyloid PET positivity thresholds in centiloids: a multisite PET study approach. Alzheimers Res Ther. 2021;13(1):99. doi:10.1186/s13195-021-00836-1 33971965 PMC8111744

[21] Cutter GR , Baier ML , Rudick RA , et al. Development of a multiple sclerosis functional composite as a clinical trial outcome measure. Brain. 1999;122:871‐882. doi:10.1093/brain/122.5.871. Pt 5. 10355672

[22] Anti‐amyloid treatment in asymptomatic Alzheimer's disease (A4 Study). A4 /LEARN Data FAQ: 2024‐2006‐27 (v1.0).

[23] Stata Statistical Software: Release 17. StataCorp LLC; 2021.

[24] Cox CG , Kirch M , Singer D , et al. Older US adults' experiences with and views about cognitive screening and blood biomarker testing for Alzheimer's disease. Alzheimers Dement (Amst). 2025;17(1):e70067. doi:10.1002/dad2.70067 39822297 PMC11736703

[25] Sperling RA , Donohue MC , Rissman RA , et al. Amyloid and tau prediction of cognitive and functional decline in unimpaired older individuals: longitudinal data from the A4 and LEARN studies. J Prev Alzheimers Dis. 2024;11(4):802‐813. doi:10.14283/jpad.2024.122 39044488 PMC11266444

[26] Chandler JM , Ye W , Mi X , Doty EG , Johnston JA . Potential impact of slowing disease progression in early symptomatic Alzheimer's disease on patient quality of life, caregiver time, and total societal costs: estimates based on findings from GERAS‐US study. J Alzheimers Dis. 2024;100(2):563‐578. doi:10.3233/JAD-231166 38875031 PMC11307086

