# Supplementary material for: Employment, volunteering, and health‐related resource use in pre‐symptomatic AD: Results from the Anti‐Amyloid Treatment in Asymptomatic Alzheimer's Disease (A4) study
Source: Alzheimers Dement. 2025 Oct 14;21(10):e70641. doi: 10.1002/alz.70641 (PMC12519503; doi:10.1002/alz.70641)
Supplement: Supplementary file 2 — Supporting file2:alz70641‐sup‐0002‐tables.docx [file ALZ-21-e70641-s002.docx]

**Supplemental Table 1. Relationship between Study Arm, PACC and RUI, including interaction effects.**

| **Variables** | **employment** |  | **volunteer** |  | **Paid help** |  | **Unpaid help** |  | **Hospitalization** |  | **Doctor visit** |  |
| --- | --- | --- | --- | --- | --- | --- | --- | --- | --- | --- | --- | --- |
| Time, months since baseline | 0.964 | ^**^ | 0.987 |  | 0.991 |  | 1.009 |  | 1.011 |  | 0.999 |  |
|  | (0.011) |  | (0.008) |  | (0.026) |  | (0.014) |  | (0.007) |  | (0.006) |  |
| Baseline group difference, A4 vs. LEARN | 2.170 | ^*^ | 0.445 | ^**^ | 0.633 |  | 0.640 |  | 0.683 | ^*^ | 0.969 |  |
|  | (0.702) |  | (0.116) |  | (0.315) |  | (0.213) |  | (0.132) |  | (0.137) |  |
| Baseline group ^*^ Time | 0.995 |  | 1.002 |  | 1.015 |  | 1.021 |  | 0.997 |  | 0.998 |  |
|  | (0.013) |  | (0.009) |  | (0.030) |  | (0.016) |  | (0.009) |  | (0.007) |  |
| Baseline PACC | 1.109 | ^+^ | 1.161 | ^**^ | 1.002 |  | 1.004 |  | 0.937 | ^*^ | 1.029 |  |
|  | (0.065) |  | (0.057) |  | (0.094) |  | (0.062) |  | (0.030) |  | (0.027) |  |
| Baseline PACC ^*^ Time | 1.000 |  | 1.000 |  | 0.996 |  | 1.000 |  | 1.001 |  | 1.000 |  |
|  | (0.002) |  | (0.001) |  | (0.004) |  | (0.002) |  | (0.001) |  | (0.001) |  |
| PACC Change from Baseline | 1.055 |  | 1.183 | ^***^ | 0.886 | ^+^ | 0.933 | ^+^ | 0.976 |  | 1.085 | ^***^ |
|  | (0.054) |  | (0.042) |  | (0.063) |  | (0.038) |  | (0.028) |  | (0.024) |  |
| Age | 0.776 | ^***^ | 1.100 | ^***^ | 1.104 | ^***^ | 1.113 | ^***^ | 1.045 | ^***^ | 1.019 |  |
|  | (0.026) |  | (0.026) |  | (0.033) |  | (0.027) |  | (0.014) |  | (0.012) |  |
| Female vs. male | 0.131 | ^***^ | 1.683 | ^*^ | 1.697 | ^+^ | 2.419 | ^***^ | 0.903 |  | 0.915 |  |
|  | (0.044) |  | (0.372) |  | (0.518) |  | (0.540) |  | (0.117) |  | (0.093) |  |
| URG vs. NHW | 5.523 | ^**^ | 0.582 |  | 0.609 |  | 1.071 |  | 0.819 |  | 0.868 |  |
|  | (3.231) |  | (0.200) |  | (0.344) |  | (0.325) |  | (0.185) |  | (0.141) |  |
| Education | 1.167 | ^**^ | 1.203 | ^***^ | 1.124 | ^*^ | 1.047 |  | 1.011 |  | 1.041 | ^*^ |
|  | (0.068) |  | (0.048) |  | (0.053) |  | (0.036) |  | (0.027) |  | (0.020) |  |
| BMI | 1.024 |  | 0.979 |  | 1.114 | ^***^ | 1.042 | ^*^ | 1.052 | ^***^ | 1.030 | ^**^ |
|  | (0.026) |  | (0.020) |  | (0.034) |  | (0.021) |  | (0.012) |  | (0.010) |  |
| Hypertension | 1.379 |  | 1.495 |  | 0.499 |  | 0.720 |  | 0.692 |  | 1.370 |  |
|  | (0.820) |  | (0.639) |  | (0.355) |  | (0.328) |  | (0.204) |  | (0.288) |  |

**Supplemental Table 2. Relationship between Study Arm, CDR and RUI, including interaction effects.**

| **Variables** | **employment** |  | **volunteer** |  | **Paid help** |  | **Unpaid help** |  | **Hospitalization** |  | **Doctor visit** |  |
| --- | --- | --- | --- | --- | --- | --- | --- | --- | --- | --- | --- | --- |
| Time, months since baseline | 0.961 | ^**^ | 0.992 |  | 0.981 |  | 1.007 |  | 1.011 |  | 1.005 |  |
|  | (0.012) |  | (0.009) |  | (0.027) |  | (0.015) |  | (0.008) |  | (0.006) |  |
| Baseline group difference, A4 vs. LEARN | 2.982 | ^**^ | 0.381 | ^**^ | 0.614 |  | 0.576 |  | 0.813 |  | 1.235 |  |
|  | (1.040) |  | (0.118) |  | (0.336) |  | (0.222) |  | (0.192) |  | (0.215) |  |
| Baseline group ^*^ Time | 0.986 |  | 1.002 |  | 1.026 |  | 1.024 |  | 0.995 |  | 0.985 | ^*^ |
|  | (0.014) |  | (0.010) |  | (0.031) |  | (0.017) |  | (0.010) |  | (0.008) |  |
| SCREENING CDR=0.5 | 0.332 | ^*^ | 0.334 | ^*^ | 0.387 |  | 1.468 |  | 0.878 |  | 1.710 | ^+^ |
|  | (0.178) |  | (0.165) |  | (0.511) |  | (0.973) |  | (0.326) |  | (0.519) |  |
| SCREENING CDR * Time | 1.010 |  | 1.006 |  | 1.037 |  | 0.989 |  | 1.011 |  | 0.991 |  |
|  | (0.022) |  | (0.015) |  | (0.047) |  | (0.024) |  | (0.014) |  | (0.011) |  |
| CDRSB Change from baseline | 0.646 | ^*^ | 0.556 | ^***^ | 1.511 | ^***^ | 1.305 | ^***^ | 1.069 |  | 1.063 |  |
|  | (0.139) |  | (0.082) |  | (0.188) |  | (0.103) |  | (0.086) |  | (0.089) |  |
| Age | 0.777 | ^***^ | 1.064 | ^*^ | 1.158 | ^***^ | 1.126 | ^***^ | 1.052 | ^***^ | 1.001 |  |
|  | (0.026) |  | (0.027) |  | (0.034) |  | (0.025) |  | (0.014) |  | (0.011) |  |
| Female vs. male | 0.152 | ^***^ | 2.114 | ^**^ | 1.698 | ^+^ | 2.081 | ^***^ | 0.928 |  | 0.898 |  |
|  | (0.050) |  | (0.513) |  | (0.524) |  | (0.424) |  | (0.119) |  | (0.095) |  |
| URG vs. NHW | 5.092 | ^**^ | 0.587 |  | 0.667 |  | 1.006 |  | 0.992 |  | 0.968 |  |
|  | (2.818) |  | (0.229) |  | (0.377) |  | (0.309) |  | (0.211) |  | (0.167) |  |
| Education | 1.227 | ^***^ | 1.238 | ^***^ | 1.157 | ^**^ | 1.064 | ^+^ | 0.978 |  | 1.035 | ^+^ |
|  | (0.074) |  | (0.056) |  | (0.055) |  | (0.037) |  | (0.025) |  | (0.020) |  |
| BMI | 1.010 |  | 0.969 |  | 1.114 | ^***^ | 1.058 | ^**^ | 1.060 | ^***^ | 1.030 | ^**^ |
|  | (0.025) |  | (0.022) |  | (0.030) |  | (0.018) |  | (0.012) |  | (0.011) |  |
| Hypertension | 0.897 |  | 1.125 |  | 0.172 | ^+^ | 0.708 |  | 1.011 |  | 1.276 |  |
|  | (0.528) |  | (0.554) |  | (0.163) |  | (0.281) |  | (0.267) |  | (0.277) |  |

**Supplemental Table 3. Relationship between Amyloid CL Tertile, PACC and RUI, including interaction effects.**

| **Variables** | **Employment** | | **Volunteer** |  | **Paid help** |  | **Unpaid help** |  | **Hospitalization** |  | **Doctor visit** |  |
| --- | --- | --- | --- | --- | --- | --- | --- | --- | --- | --- | --- | --- |
|  | OR (SE) |  | OR (SE) |  | OR (SE) |  | OR (SE) |  | OR (SE) |  | OR (SE) |  |
| Time, months since baseline | 0.964 | ^***^ | 0.993 |  | 0.987 |  | 1.025 | ^**^ | 1.011 | ^*^ | 0.999 |  |
|  | (0.008) |  | (0.005) |  | (0.018) |  | (0.009) |  | (0.005) |  | (0.004) |  |
| Baseline Amyloid CL  (reference=lowest tertile) |  |  |  |  |  |  |  |  |  |  |  |  |
| Amyloid CL 6.1-77.2 | 1.661 |  | 0.845 |  | 0.579 |  | 0.853 |  | 0.814 |  | 1.090 |  |
|  | (0.614) |  | (0.246) |  | (0.324) |  | (0.311) |  | (0.181) |  | (0.175) |  |
| Amyloid CL > 77.2 | 1.287 |  | 0.764 |  | 0.733 |  | 0.721 |  | 0.862 |  | 0.829 |  |
|  | (0.448) |  | (0.225) |  | (0.385) |  | (0.275) |  | (0.192) |  | (0.132) |  |
| Interaction between group x time |  |  |  |  |  |  |  |  |  |  |  |  |
| Amyloid CL 6.1-77.2 | 0.998 |  | 0.995 |  | 1.035 |  | 0.989 |  | 1.000 |  | 0.989 |  |
|  | (0.013) |  | (0.008) |  | (0.026) |  | (0.014) |  | (0.009) |  | (0.007) |  |
| Amyloid CL > 77.2 | 0.986 |  | 0.992 |  | 1.021 |  | 1.011 |  | 0.990 |  | 1.007 |  |
|  | (0.014) |  | (0.009) |  | (0.028) |  | (0.014) |  | (0.010) |  | (0.007) |  |
| Baseline PACC | 1.098 |  | 1.171 | ^**^ | 1.003 |  | 1.002 |  | 0.943 | ^+^ | 1.023 |  |
|  | (0.064) |  | (0.058) |  | (0.091) |  | (0.062) |  | (0.031) |  | (0.027) |  |
| Baseline PACC ^*^ Time | 1.000 |  | 1.000 |  | 0.996 |  | 1.000 |  | 1.001 |  | 1.000 |  |
|  | (0.002) |  | (0.001) |  | (0.004) |  | (0.002) |  | (0.001) |  | (0.001) |  |
| PACC Change from baseline | 1.044 |  | 1.179 | ^***^ | 0.892 |  | 0.932 | ^+^ | 0.974 |  | 1.089 | ^***^ |
|  | (0.053) |  | (0.042) |  | (0.064) |  | (0.039) |  | (0.029) |  | (0.024) |  |
| Age | 0.779 | ^***^ | 1.097 | ^***^ | 1.103 | ^**^ | 1.113 | ^***^ | 1.045 | ^**^ | 1.020 | ^+^ |
|  | (0.027) |  | (0.027) |  | (0.033) |  | (0.027) |  | (0.014) |  | (0.012) |  |
| Female vs. male | 0.133 | ^***^ | 1.705 | ^*^ | 1.696 | ^+^ | 2.365 | ^***^ | 0.907 |  | 0.915 |  |
|  | (0.045) |  | (0.380) |  | (0.529) |  | (0.524) |  | (0.118) |  | (0.094) |  |
| URG vs. NHW | 5.494 | ^**^ | 0.576 |  | 0.615 |  | 1.048 |  | 0.804 |  | 0.858 |  |
|  | (3.242) |  | (0.202) |  | (0.352) |  | (0.320) |  | (0.184) |  | (0.141) |  |
| Education | 1.167 | ^**^ | 1.205 | ^***^ | 1.125 | ^*^ | 1.045 |  | 1.012 |  | 1.041 | ^*^ |
|  | (0.069) |  | (0.049) |  | (0.054) |  | (0.036) |  | (0.027) |  | (0.020) |  |
| BMI | 1.025 |  | 0.978 |  | 1.113 | ^***^ | 1.040 | ^+^ | 1.051 | ^***^ | 1.029 | ^**^ |
|  | (0.026) |  | (0.020) |  | (0.033) |  | (0.021) |  | (0.012) |  | (0.010) |  |
| Hypertension | 1.361 |  | 1.502 |  | 0.505 |  | 0.729 |  | 0.701 |  | 1.368 |  |
|  | (0.811) |  | (0.643) |  | (0.352) |  | (0.330) |  | (0.212) |  | (0.288) |  |

**Supplemental Table 4. Relationship between Amyloid CL Tertile, CDR, and RUI, including interaction effects**

| **Variables** | **Employment** | | **Volunteer** |  | **Paid help** |  | **Unpaid help** |  | **Hospitalization** |  | **Doctor visit** |  |
| --- | --- | --- | --- | --- | --- | --- | --- | --- | --- | --- | --- | --- |
|  | OR (SE) |  | OR (SE) |  | OR (SE) |  | OR (SE) |  | OR (SE) |  | OR (SE) |  |
| Time, months since baseline | 0.963 | ^***^ | 0.992 |  | 0.984 |  | 1.019 | ^+^ | 1.009 |  | 0.997 |  |
|  | (0.009) |  | (0.007) |  | (0.019) |  | (0.010) |  | (0.006) |  | (0.005) |  |
| Baseline Amyloid CL  (reference=lowest tertile) |  |  |  |  |  |  |  |  |  |  |  |  |
| Amyloid CL 6.1-77.2 | 3.425 | ^**^ | 0.747 |  | 0.782 |  | 1.158 |  | 1.165 |  | 1.404 |  |
|  | (1.557) |  | (0.291) |  | (0.591) |  | (0.559) |  | (0.382) |  | (0.340) |  |
| Amyloid CL > 77.2 | 2.254 | ^+^ | 0.514 | ^+^ | 1.027 |  | 0.255 | ^*^ | 1.000 |  | 0.782 |  |
|  | (0.995) |  | (0.206) |  | (0.678) |  | (0.150) |  | (0.315) |  | (0.189) |  |
| Interaction between group x time |  |  |  |  |  |  |  |  |  |  |  |  |
| Amyloid CL 6.1-77.2 | 0.975 |  | 1.002 |  | 1.029 |  | 0.985 |  | 0.990 |  | 0.983 | ^*^ |
|  | (0.016) |  | (0.012) |  | (0.031) |  | (0.018) |  | (0.012) |  | (0.009) |  |
| Amyloid CL > 77.2 | 0.975 |  | 0.998 |  | 1.018 |  | 1.040 | ^*^ | 0.993 |  | 1.003 |  |
|  | (0.017) |  | (0.012) |  | (0.027) |  | (0.020) |  | (0.012) |  | (0.009) |  |
| SCREENING CDR=0.5 | 0.327 | ^*^ | 0.336 | ^*^ | 0.341 |  | 1.503 |  | 0.865 |  | 1.733 | ^+^ |
|  | (0.177) |  | (0.167) |  | (0.458) |  | (1.018) |  | (0.323) |  | (0.534) |  |
| SCREENING CDR * Time | 1.009 |  | 1.006 |  | 1.040 |  | 0.989 |  | 1.012 |  | 0.991 |  |
|  | (0.022) |  | (0.015) |  | (0.049) |  | (0.025) |  | (0.014) |  | (0.011) |  |
| CDRSB Change from baseline | 0.666 | ^+^ | 0.569 | ^***^ | 1.482 | ^**^ | 1.302 | ^**^ | 1.079 |  | 1.059 |  |
|  | (0.147) |  | (0.084) |  | (0.186) |  | (0.111) |  | (0.091) |  | (0.089) |  |
| Age | 0.776 | ^***^ | 1.063 | ^*^ | 1.150 | ^***^ | 1.129 | ^***^ | 1.051 | ^***^ | 1.002 |  |
|  | (0.027) |  | (0.027) |  | (0.035) |  | (0.025) |  | (0.014) |  | (0.012) |  |
| Female vs. male | 0.147 | ^***^ | 2.172 | ^**^ | 1.702 | ^+^ | 2.047 | ^***^ | 0.932 |  | 0.898 |  |
|  | (0.050) |  | (0.532) |  | (0.535) |  | (0.413) |  | (0.120) |  | (0.095) |  |
| URG vs. NHW | 5.266 | ^**^ | 0.568 |  | 0.698 |  | 0.974 |  | 0.985 |  | 0.952 |  |
|  | (2.976) |  | (0.226) |  | (0.396) |  | (0.300) |  | (0.212) |  | (0.165) |  |
| Education | 1.228 | ^***^ | 1.241 | ^***^ | 1.157 | ^**^ | 1.063 | ^+^ | 0.979 |  | 1.034 | ^+^ |
|  | (0.075) |  | (0.056) |  | (0.056) |  | (0.038) |  | (0.025) |  | (0.020) |  |
| BMI | 1.012 |  | 0.968 |  | 1.115 | ^***^ | 1.057 | ^**^ | 1.059 | ^***^ | 1.029 | ^**^ |
|  | (0.025) |  | (0.022) |  | (0.030) |  | (0.018) |  | (0.012) |  | (0.011) |  |
| Hypertension | 0.880 |  | 1.132 |  | 0.171 | ^+^ | 0.703 |  | 1.023 |  | 1.273 |  |
|  | (0.521) |  | (0.561) |  | (0.159) |  | (0.281) |  | (0.274) |  | (0.276) |  |
